# Hodgkin lymphoma and Ewing sarcoma in pediatric patient carrying germline *PALB2* variant: a case report and literature review

**DOI:** 10.3389/fonc.2025.1514697

**Published:** 2025-02-18

**Authors:** Jakub Czarny, Dominika Galli, Agnieszka Wziątek, Agata Pastorczak, Bartosz Szmyd, Borys Przybyszewski, Anna Raciborska, Katarzyna Jończyk-Potoczna, Katarzyna Derwich

**Affiliations:** ^1^ Faculty of Medicine, Poznan University of Medical Sciences, Poznan, Poland; ^2^ Department of Pediatric Oncology, Hematology and Transplantology, Institute of Pediatrics, Poznan University of Medical Sciences, Poznan, Poland; ^3^ Department of Pediatrics, Oncology and Hematology, Medical University of Lodz, Lodz, Poland; ^4^ Department of Oncology and Surgical Oncology for Children and Youth, Institute of Mother and Child, Warsaw, Poland; ^5^ Department of Pediatric Radiology, Institute of Pediatrics, Poznan University of Medical Sciences, Poznan, Poland

**Keywords:** PALB2, Hodgkin lymphoma, Ewing’s sarcoma, genetic predisposition, tumorigenesis

## Abstract

Germinal predisposition to malignancy is found in approximately 10% of oncological pediatric patients. As awareness of cancer risk factors associated with germline mutations increases, and with advancements in molecular techniques, more carefully selected patients are being tested. This approach enables the identification of new variants—both those that are clearly linked to tumorigenesis and candidates, which biological role needs to be functionally verified. Pathogenic variants within cancer-predisposing genes not only increase nearly eightfold the risk of secondary cancers but also may be associated with excessive toxicity of antineoplastic treatment. We present the case of a girl who developed classical Hodgkin lymphoma at the age of 8 years and secondary Ewing sarcoma at the age of 16 years. Her father was diagnosed with classical Hodgkin lymphoma at the age of 27 years. Genetic testing revealed the carriership of a germline heterozygous variant in the *PALB2* gene (NM_024675.4:c.110G>A, p.Arg37His) in both the patient and her father. Since the patient was exposed to chemotherapy due to lymphoma prior to the development of secondary malignancy and the variant is classified as an aberration of unknown significance, the causative role of the *PALB2* variant remains uncertain. Nevertheless, the presented case may indicate the possible interplay between inherited genetic predisposition and the exposure to cytostatic drugs, which both are involved in promoting secondary cancers in pediatric patients.

## Introduction

Germline predisposition to malignancies concerns approximately 10% of childhood cancer patients (1–3). However, due to rising awareness, development of molecular techniques, and their broad application to the clinical testing, the role of inborn susceptibility to malignancies in children is recognized as a significant factor promoting the development of childhood neoplasms. Pediatric patients are selected for testing of genetic predisposition to cancer using defined clinical criteria indicating on the increased genetic susceptibility to malignancy. This includes at least two malignancies in childhood (4, 5), at least one first-degree and/or two second-degree (on the same side of the family) patients with cancer under 45 years, bilateral neoplasms, parental consanguinity, specific histological types of malignancies particularly associated with germline genetic defects, excessive toxicities of oncological treatment, comorbidities (congenital anomalies, facial dysmorphisms, intellectual disability, aberrant growth, skin anomalies, hematological disorders, or immune deficiency), and secondary malignancies during childhood. Such probands require complex genetic testing using appropriate next-generation sequencing (NGS) panels, which will unravel potential causes of carcinogenesis (2, 6). The diagnosis of genetic predisposition to malignancy is beneficial both for the child with already diagnosed malignancy, as well as for their families. First, it enables introduction of the oncological surveillance and early detection of secondary neoplasms, as well as predicting potential increased toxicity and resistance to standard treatment and modifying treatment schedules. Patients’ family members may benefit from genetic counselling, oncological screening, early malignancy detection, reproductive counseling, and proper prenatal diagnosis (2, 6).

We present the case of a pediatric patient who developed Hodgkin lymphoma at the age of 8 years and secondary Ewing sarcoma at the age of 16 years with a positive family history of cancer, who carries a germline heterozygous variant of unknown significance in the *PALB2* gene.

## Case report

The 8-year-old female patient was referred to the pediatric oncology clinic due to cervical lymphadenopathy concurring with pharyngitis and B symptoms. The birth history was unremarkable. The patient does not present any dysmorphic features, as well as immune deficiencies, both at laboratory and clinical levels.

**Figure 1:**
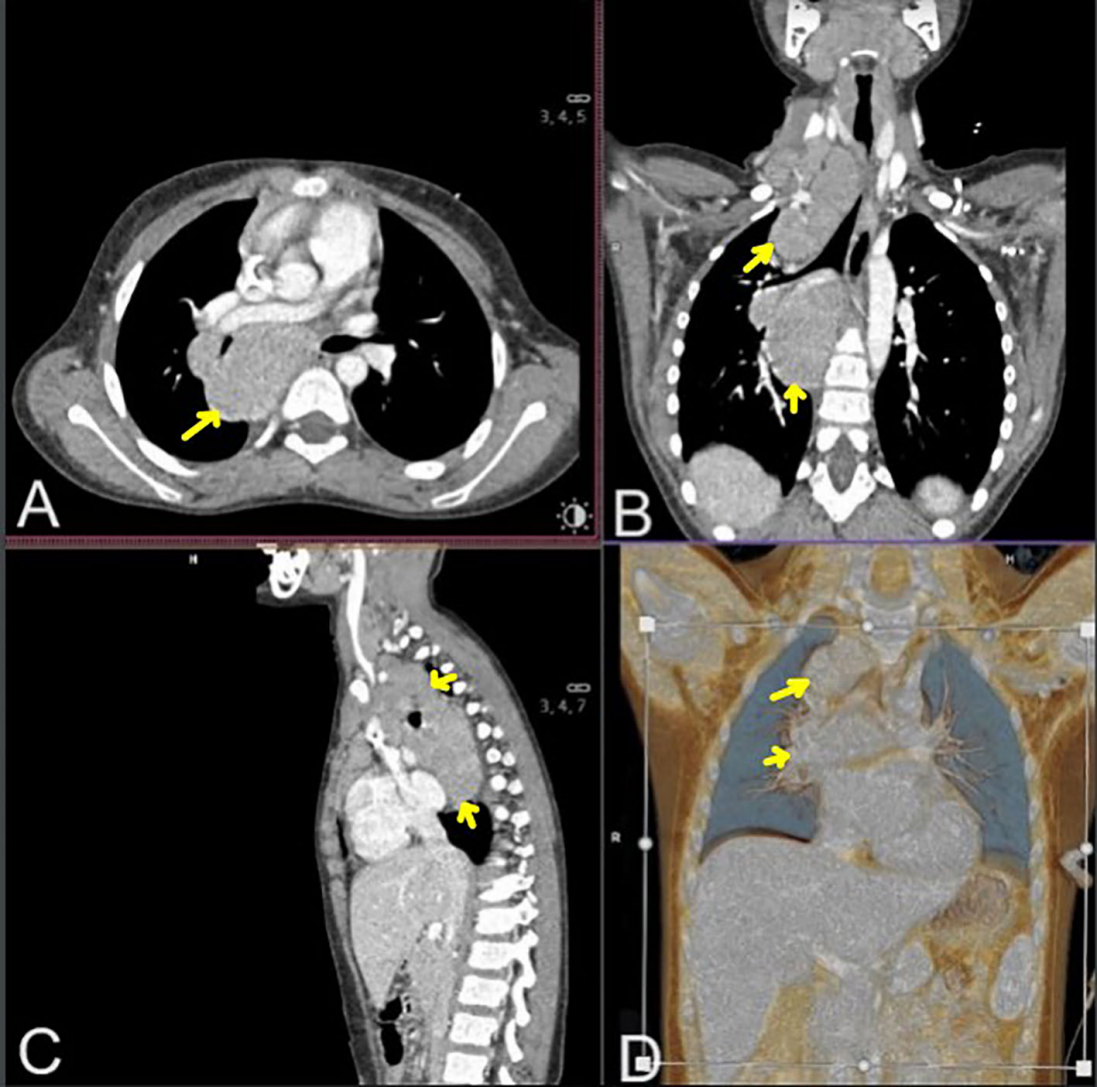
Chest and neck computed tomography scans in mediastinal window after contrast agent administration: transverse **(A)**, coronal [**(B)** multiplanar reconstruction, **(D)** volume rendering technique reconstruction], and sagittal **(C)** section. Neck and mediastinal Hodgkin lymphoma masses are marked with yellow arrows.

During the diagnostic process, chest computed tomography (CT) revealed a well-defined, heterogeneous mediastinal tumor measuring 10.8 cm × 6.0 cm × 5.3 cm (cc × ap × ds), exhibiting contrast enhancement. The tumor adhered to the anterior chest wall, extending from the superior thoracic aperture to the level of Th8. It was in communication with the enlarged cervical lymph nodes and involved the right lung cavity without enlargement of the left hilar lymph nodes (**Figure 1**). Abdominal CT revealed multiple hypodense focal lesions in the spleen. Positron emission tomography (PET) CT confirmed a metabolically active conglomerate of right neck lymph nodes, a mediastinal tumor, as well as increased uptake in the spleen lesions.

A lymph node biopsy was performed. Finally, the patient was diagnosed with classical Hodgkin lymphoma, nodular sclerosis type, IIIB grade, and was qualified to the therapeutic group TL-3 according to the EuroNET-PHL-C1 treatment protocol.

The patient was administered two chemotherapy cycles of OEPA. Due to the adequate response according to the protocol, she received four cycles of COPDAC-28 and did not require radiotherapy. Complete remission was achieved. The patient was remaining under the care of the outpatient children’s oncology clinic.

**Figure 2:**
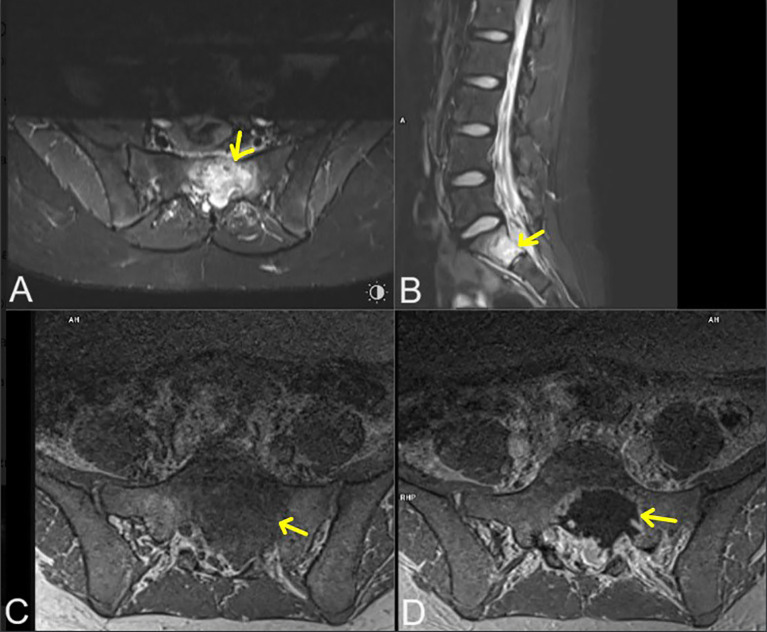
Lumbosacral magnetic resonance imaging in: T2-weighted (STIR) axial projection **(A)**, T2-weighted (STIR) sagittal **(B)**, T1-weighted (vibe) axial **(C)**, T1-weighted (vibe) axial with contrast agent administration **(D)**. Metastatic lesions marked with yellow arrows.

**Figure 3:**
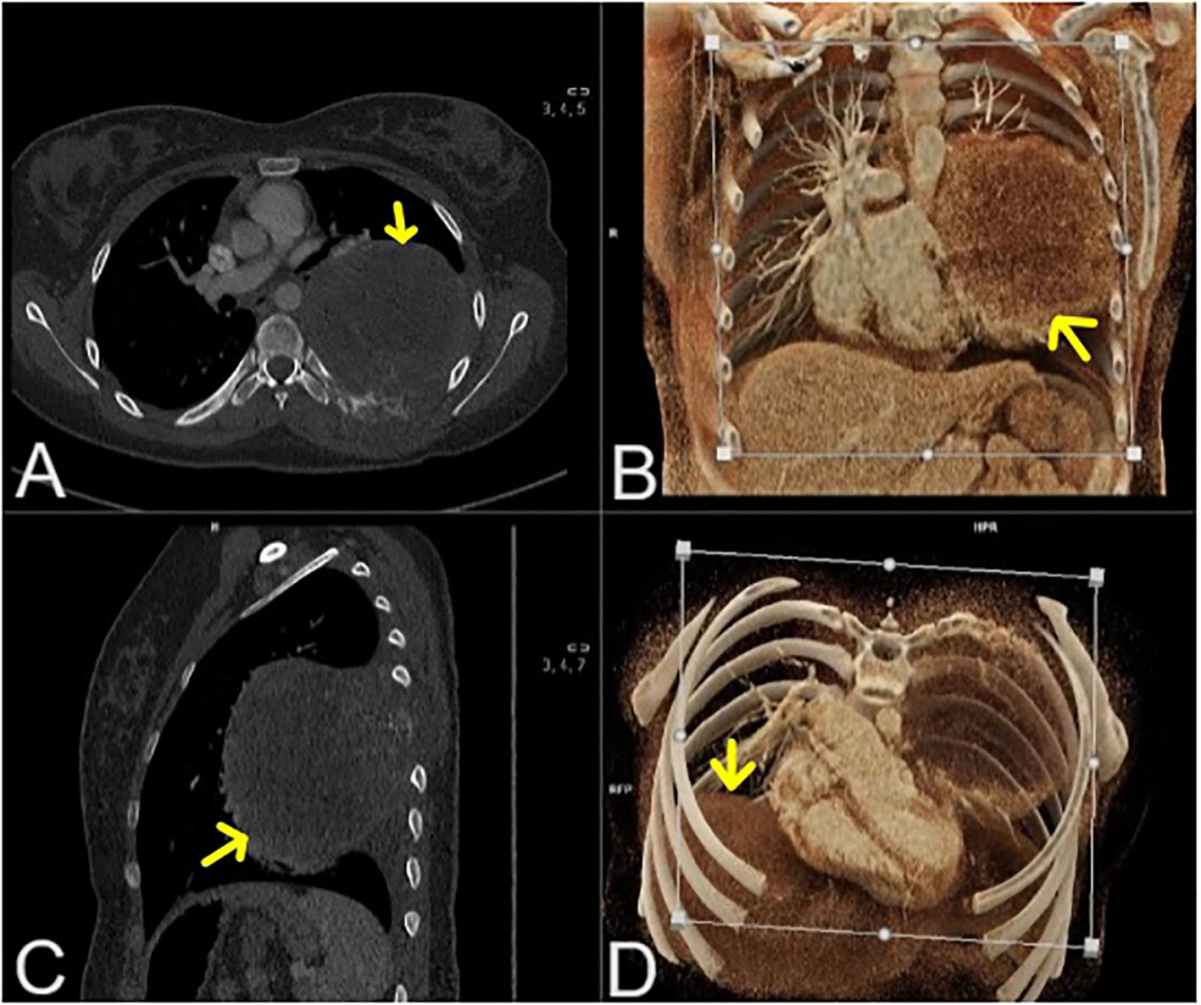
Chest computed tomography (mediastinal window after contrast agent administration- transverse [multiplanar reconstruction **(A)**, volume rendering technique reconstruction **(D)**], coronal [volume rendering technique reconstruction **(B)**] and sagittal [multiplanar reconstruction **(C)** section]. Primary tumor mass marked with yellow arrows.

Five years after the protocol completion, at the age of 16 years, she started complaining of leg pain and lower back pain. Magnetic resonance imaging (MRI) of the lumbosacral spine revealed a well-defined lesion in the S1 vertebral body, located medially on the left side, measuring 38 mm × 20 mm × 22 mm (ds × ap × cc). Within the spinal canal, compression of the cauda equina threads and segmental destruction of the cortical layer of the anterior surface of the vertebral body were visible (**Figure 2**). Thoracic CT revealed a well-defined, round, solid lesion measuring 10 cm × 10 cm × 11 cm (ds × ap × cc) on the left side, located medially, paravertebrally, and along the posterior-lateral chest wall. The lesion appeared to potentially originate from the sixth left rib. At the level of the lesion, the image showed destruction (osteolysis, destruction of the cortical layer, and swelling) of the posterior segment of the sixth left rib over a length of about 6 cm. The lesion penetrated the adjacent intercostal spaces at this level and intercostal muscles. Medially, the tumor adhered to the spine, causing a slight curvature of the spine to the right side with penetration into the C6/C7 intervertebral foramen. The tumor caused the displacement of mediastinal structures to the right side with forward displacement and narrowing of the left main bronchus (**Figure 3**). Moreover, after the second cycle of chemotherapy, three-phase scintigraphy of the skeletal system with 99mTc-MDP of activity 13 mCi revealed in the phase III whole-body and thoracic SPECT and pelvic-targeted SPECT/CT increased tracer accumulation also in the upper lateral part of the L3 vertebral body on the right side, in the head of the left femur, as well as in the projection of the distal femoral epiphyses and proximal tibial epiphysis with marked asymmetry (left over right).

A biopsy of the chest wall tumor was performed and Ewing sarcoma was detected. Bone marrow aspiration biopsy and trepanobiopsy excluded bone marrow involvement.

She underwent preoperative chemotherapy cycles of vincristine-doxorubicin-cyclophosphamide (the fourth adjuvant cycle did not include doxorubicin due to the planned radiotherapy) and ifosfamid-etopophos (due to an allergic reaction to etoposide). After the fourth cycle of chemotherapy, VMAT radiotherapy was administered to the area of metastatic lesions in the sacrum, L3 vertebra, and left femur at a total dose of 3,600 cGy. Preoperative imaging tests (MRI, PET-CT) showed a good response to the induction therapy. Subsequently, a resection of the tumor was performed (the sixth rib and part of the seventh rib on the left side were removed). Next, she continued the adjuvant chemotherapy, as well as VMAT breast-preserving radiotherapy, which was distributed to the lodge of the tumor with the margin at a dose of 4,500 cGy. Currently, the patient remains in complete remission under the care of the pediatric oncology outpatient clinic.

In the biopsy material, FoundationOne^®^ CDx NGS panel detected *EWSR1::FLI1* fusion and the following sequence variants: in *PALB2* gene (p.Arg37His, in *SGK1* gene (p.Ala40fs*21), in *TSC2* gene (p.Ala357Val), in *MRE11A* gene (p.Ile120Val), in *PIM1* gene (p.Glu124Gln), in *RPTOR* gene (p.Ala862Thr). No tumor mutational burden and microsatellite instability were revealed.

**Figure 4:**
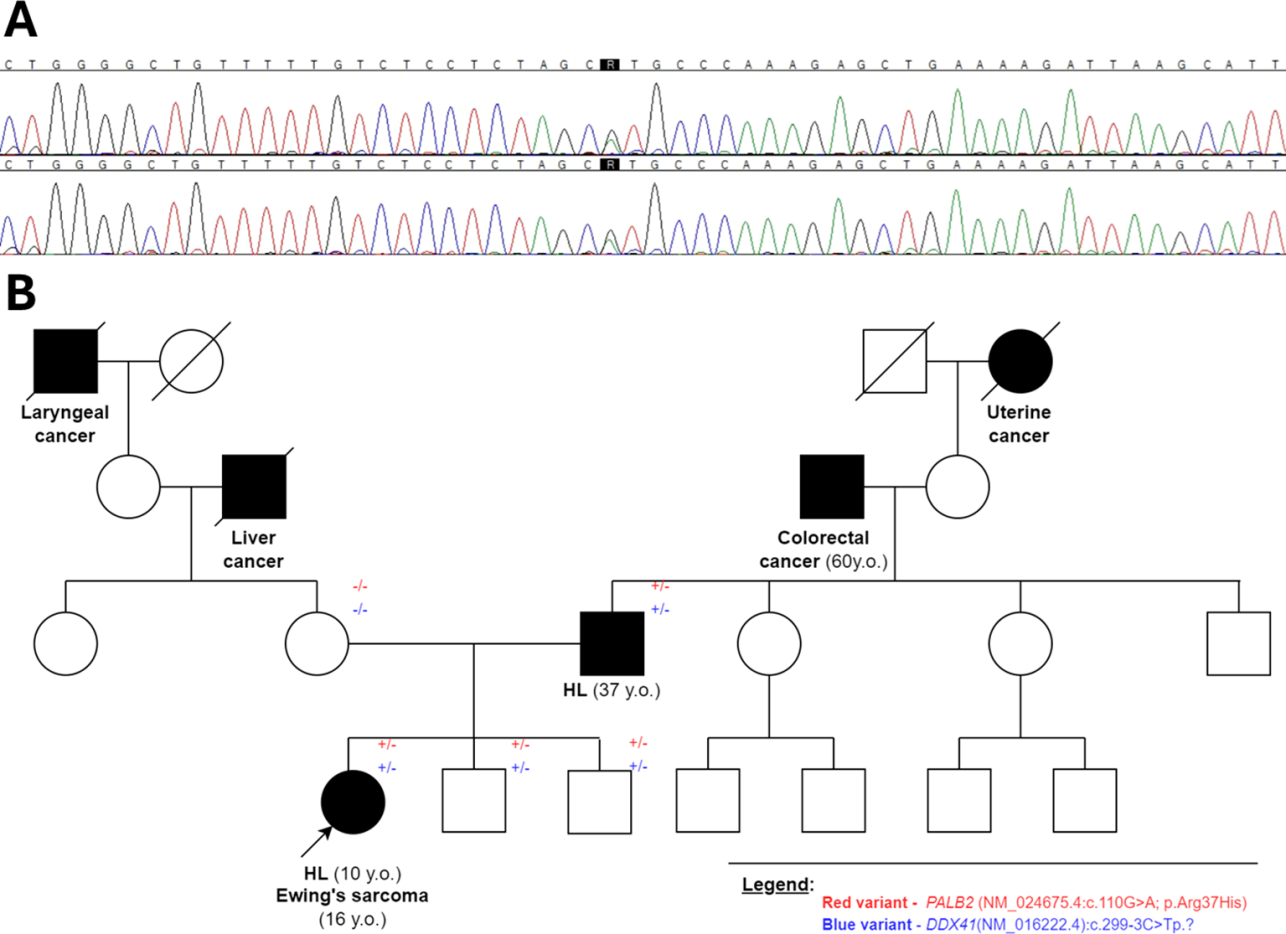
**(A)** Chromatogram confirming the presence of heterozygous missense variant (NM_024675.4):c.110G>A;p.Arg37His within the *PALB2* gene sequence in the child and the father; **(B)** the pedigree showing the carriership of identified variants in proband’s family members.

Given the family’s positive history of lymphoma, the patient was offered genetic testing for genetic predisposition to malignancy. We performed trio exome sequencing using germinal DNA isolated from peripheral blood collected at remission from the patient and her parents. The analysis was performed using the hg19 reference genome and the DRAGEN Germline Pipeline (basespace.com). The variants, which are shared only by the child and the father, were firstly filtered based on a list of genes related to childhood cancer risk and associated with immunological and/or hematological abnormalities. We analyzed only variants observed in more than seven, which were rarer than 5% in the obtained databases. We identified two possibly causative heterozygous alterations in proband and father: *PALB2* (NM_024675.4:c.110G>A; p.Arg37His) and *DDX41* (NM_016222.4:c.299-3C>Tp.)? (**Figure 4**, **Supplementary Table S1**). As the variant in *DDX41* could probably lead to lymphoproliferation (7) through affecting splice junctions, we assessed splicing based on patients’ mRNA, but the effect on splicing was not proved. Therefore, in the further analysis, we focused on the *PALB2* variant. Bioinformatic analysis showed its rarity—it was observed that only 10 alleles were observed among 251,458 total alleles, according to gnomAD Exomes Version: 2.1.1 encompassing the following subpopulations comprise: African/African-American, Remaining, Admixed American, European (non-Finnish), South Asian, European (Finnish), Ashkenazi Jewish, and East Asian. Remarkably, there were no reported homozygous cases for this variant, further underscoring its uncommon nature (GnomAD and ExAC databases). Furthermore, the variant’s conservation score, as assessed by both PhastCons100way (1.000) and PhyloP100way (4.318), points to its biological significance. Clinical relevance is underscored by the analysis of ClinVar submissions, where 11 of 12 entries related to hereditary cancer syndromes classified the variant as a Variant of Uncertain Significance (VUS), while only one submission suggested a likely benign nature. The majority of these reports are grounded in clinical assessments of germline DNA. *In-silico* predictions showed a nuanced view. Two predictors (DANN and SIFT) support a pathogenic interpretation, while a significant number indicate uncertain significance, and 10 suggest benign or moderately supportive evidence of benignity (see **Supplementary Table S1** for further information). Conversely, meta-analyses of *in-silico* predictors, which integrate evidence across various computational tools, lean toward a benign classification, with all assessed meta-scores offering moderate or supportive evidence for a benign interpretation (see **Supplementary Table S1**). Familial segregation analysis revealed that both variants were present not only in the patient with lymphoma but also in two of the proband’s brothers (currently at the ages 6 and 9 years), who have no history of neoplasms to date after the evaluation of the pediatric oncologist. This could be attributed to the variable degree of penetrance and the relatively young age of these boys. Considering these diverse and sometimes conflicting findings, we conducted a comprehensive literature review to further explore the implications of these variants, which is discussed in detail in the subsequent sections of this paper.

Considering all of the above, according to the ACMG classification (8), we observed one strong pathogenic criterion and two supporting ones, along with one moderate benign criterion and two supporting benign criteria. This combination results in a total score of two points, classifying the variant as one of uncertain significance (see **Table 1** for further details).

**Table 1:** The classification of variant significance according to the American College of Medical Genetics Criteria.

Criterion	Comments
PS3 (Strong)	Functional studies show a damaging effect on the gene or protein
PM2 (Supporting)	GnomAD genomes homozygous allele count = 0 is less than 2 for AD/AR gene *PALB2*, good gnomAD genomes coverage = 30.2. GnomAD exomes homozygous allele count = 0 is less than 2 for AD/AR gene *PALB2*, good gnomAD exomes coverage = 88.2.
PP4 (Supporting)	Patient phenotype or family history is particular for a disease with a single genetic cause
BP4 (Moderate)	MetaRNN = 0.255 is between 0.108 and 0.267
BP1 (Supporting)	46 out of 68 non-VUS missense variants in gene *PALB2* are benign = 67.6% which is more than the threshold of 56.9%
BP6 (Supporting)	Combined evidence strength is Supporting (score = 1). Supporting: UniProt Variants classifies this variant as Benign, citing 28319063

Scoring: 2 points = 6P − 4B ≥ Variant of uncertain significance.

## Discussion

We describe the patient who suffered from Hodgkin lymphoma and met two criteria of inclusion to testing for inherited cancer predisposition: diagnosis of secondary Ewing sarcoma during childhood and positive family history of cancer (4, 5, 9, 10). The patient is a carrier of a germline variant of uncertain significance within the *PALB2* gene. Although variants in this gene are not widely linked to increased lymphoma risk, its dysfunction, according to functional studies, may affect DNA repair and therefore promote tumorigenesis (11). In fact, genetic susceptibility to lymphomas is associated with various inborn errors, especially in primary immunodeficiencies and DNA repair disorders. Regarding genetic aberrations predisposing to sarcomas, the American College of Medical Genetics indicates the need of genetic testing among patients who developed non-Ewing sarcomas during childhood, in case of the incidence of sarcoma and other Li-Fraumeni syndrome-associated tumors in one family member or two close relatives at the age up to 45 years (8). However, some studies have reported that heterozygous pathogenic or potentially pathogenic germline mutations in DNA repair genes, for example, *FANCC*, *FANCA*, *ERCC2*, and *BRCA1*, may contribute to Ewing sarcomas because they enable the occurrence of DNA breaks leading to oncogenic gene fusions, such as *EWSR1::ETS* (12–15). Moreover, germline mutations in the *CHEK2* gene were overrepresented in some cohorts of patients diagnosed with Ewing sarcoma (13, 16). In addition, Qin et al. revealed that mutations involving homologous recombination (HR) genes are associated with an increased risk of subsequent sarcoma after treatment with alkylating agents in the third tertile (17). Our patient was administered dacarbazine and cyclophosphamide due to Hodgkin lymphoma, which could have a significant impact on the development of secondary malignancy.

PALB2 (also known as partner and localizer of BRCA2 or FANCN) is crucial for DNA repair through HR, collaborating with key effectors like BRCA1, BRCA2, RAD51, and RAD51C at DNA damage sites. Disruption of PALB2’s structure leads to genomic instability by impairing HR repair, thereby elevating the tumor mutational burden (18–21). Thus, PALB2 functions as a tumor suppressor. Heterozygous germline variants in *PALB2* significantly increase the risk for various cancers, including breast (22–32), ovarian (22, 24, 28, 31), pancreatic (22, 24, 30, 33), prostate (22, 34), colorectal (35) cancer, and so forth, with a significant decrease in these patients’ survival (34). Moreover, studies show a good response to the treatment of HR deficient (including *PALB2*-mutated tumors) with PARP inhibitors and platinum-based chemotherapy (21, 36–42). *PALB2* gene variants with defective HR are associated with higher sensitivity to cisplatin and PARP inhibitors (40). The role of *PALB2* variants in the pathogenesis of sarcomas has not been proved. However, *PALB2* variant has been observed in the patient treated due to sarcoma who also carried the variants within *PALB2* and *MITF* genes (43). This case highlights again that adult-onset cancer predisposition genes germline variants may possibly influence the presence of pediatric-onset neoplasms.

The variant detected in our patient is localized at the second exonic base from the 3’ site (44). It was previously reported in a Spanish family with non-BRCA1/BRCA2 early breast/ovarian cancer family history and the incidence of pancreatic cancer (44), as well as in a patient with unilateral breast cancer from the WECARE study (11). The functional analysis of this missense variant classified it as probably damaging, altering protein function. Two of the three prediction programs considered the variant as deleterious. The HR activity of PALB2 was proved to be impaired in this case but without the confirmed reduction of the PALB2-BRCA1 interaction. However, the decrease in the score predicted by the algorithms, the lack of variations near cryptic splice sites, and RNA analysis suggest a variant as a variant without a significant impact on the splicing process (43, 44). The variant may influence PALB2 function, therefore exacerbating DNA repair and changing the potential cancer risk after the exposure to cytostatic drugs (11, 42).

Notably, a heterozygous pathogenic *PALB2* variant has been documented in cases of acute lymphoblastic leukemia in a 6-year-old patient diagnosed with acute lymphoblastic leukemia and secondary Ewing sarcoma at the age of 12 years, originating from a family with a notable history of cancer across both paternal and maternal lineages. Genetic analysis revealed a heterozygous constitutional deletion within the *PALB2* gene (NM_024675.3:c.(1684 + ?1685-)?(2586 +?_2587-)?del). This deletion encompassed entire exons 5 and 6, leading to the premature termination of mRNA translation or the production of a truncated, dysfunctional PALB2 protein. Unfortunately, information on the familial segregation of this variant remains unavailable, precluding further insights into its hereditary transmission and phenotypic consequences of the variant (45).

Our findings reveal a variant situated at the second exonic base from the 3’ end. Initial functional analysis indicates that the R37H variant diminishes PALB2’s HR activity. This reduction suggests potential disruption to the coiled-coil motif’s integrity, implying that even minimal structural distortions could impair HR activity without necessarily affecting BRCA1 binding (11). Remarkably, this variant is the first to demonstrate a relative HR efficiency below 50%, significantly higher than the ~10% or less efficiency observed in truncating variants (46). The penetrance of this genetic variant and its role in phenotype severity remain poorly understood, necessitating further study. However, the observed decrease in HR efficiency is associated with an enhanced response to PARP inhibitors, suggesting potential meaning for therapeutic intervention (47).

Finally, it should be mentioned that the discussed variant was observed in two brothers without cancer. They are currently 6 and 9 years old, which means they are younger than their sister at the time of her cancer development and significantly younger than their father, who developed HL at the age of 37. In the further analysis, we have to consider not only age-related cancer risk but also hormonal factors (cancer risk can be influenced by hormonal changes or developmental processes that occur later in life; the brothers may not yet have reached this stage), possibly environmental factors, as well as the impact of modifier genes (the brothers may carry protective genetic variants in other genes that modulate the effects of the *PALB2* variant, reducing their overall cancer susceptibility) juxtaposed with the impact of epigenetic regulation.

## Conclusion

The reported patient exemplifies the possible interplay between germline genetic predisposition to cancer and exposure to chemical compounds in promoting secondary tumors during childhood. It suggests that the causative role of variants in adult cancer-predisposing genes in the development of pediatric malignancies needs to be functionally studied.

## Data Availability

The datasets presented in this study can be found in online repositories. The names of the repository/repositories and accession number(s) can be found in the article/**Supplementary Material**.
