# Supplementary material for: Hodgkin lymphoma and Ewing sarcoma in pediatric patient carrying germline PALB2 variant: a case report and literature review
Source: Front Oncol. 2025 Feb 18;15:1514697. doi: 10.3389/fonc.2025.1514697 (PMC11876119; doi:10.3389/fonc.2025.1514697)
Supplement: Supplementary file 1 [file DataSheet1.pdf]

Variant	*PALB2*(NM_024675.4):c.110G>A, p.Arg37His; heterozygous		
**Frequency**			
**gnomAD genomes**	0.0000319		
**gnomAD exomes**	0.0000398		
**Conservation**			
**Scores**	**Value**		**Version**
**PhastCons100way**	1.000		version 14-Apr-2021
**PhyloP100way**	4.318		version 13-Apr-2021
**Pathogenicity Scores**			
**Meta scores**			
**Engine**	**Calibrated Prediction**	**Score**	**Version**
BayesDel addAF	Benign Supporting	-0.0464	dbNSFP version 4.5
MetaLR	Benign Moderate	0.0819	dbNSFP version 4.5
MetaRNN	Benign Moderate	0.2548, 0.2548	dbNSFP version 4.5
MetaSVM	Benign Moderate	-0.8035	dbNSFP version 4.5
BayesDel noAF	Benign Supporting	-0.1229	dbNSFP version 4.5
REVEL	Benign Moderate	0.158	dbNSFP version 4.5
**Individual Predictions**			
dbscSNV	Benign Moderate	0.1525	version v1.1
MaxEntScan	Benign Moderate	0.4283	version 5-Apr-2023
MutationTaster	Benign Moderate	0.623	dbNSFP version 4.5
DANN	Pathogenic Supporting	0.9993	version 2014
SIFT	Pathogenic Supporting	0	dbNSFP version 4.5
FATHMM	Benign Supporting	1.73, 0.4	dbNSFP version 4.5
FATHMM-XF	Benign Supporting	0.511	dbNSFP version 4.5
M-CAP	Benign Supporting	0.03925	dbNSFP version 4.5
MVP	Benign Supporting	0.7812, 0.7812	dbNSFP version 4.5
BLOSUM	Uncertain	-1	version BLOSUM100
CADD	Uncertain	24.3999	version 1.6
DEOGEN2	Uncertain	0.479, 0.1732	dbNSFP version 4.5
EIGEN	Uncertain	0.3707	dbNSFP version 4.5
EIGEN PC	Uncertain	0.3411	dbNSFP version 4.5
FATHMM-MKL	Uncertain	0.9142	dbNSFP version 4.5
LIST-S2	Uncertain	0.9109, 0.9075	dbNSFP version 4.5
LRT	Uncertain	0.000148	dbNSFP version 4.5
Mutation assessor	Uncertain	2.725	dbNSFP version 4.5
PrimateAI	Uncertain	0.5256	dbNSFP version 4.5
PROVEAN	Uncertain	-3.46	dbNSFP version 4.5
SIFT4G	Uncertain	0.009	dbNSFP version 4.5
